# Supplementary material for: Predicting visual function by interpreting a neuronal wiring diagram
Source: Nature. 2024 Oct 2;634(8032):113–23. doi: 10.1038/s41586-024-07953-5 (PMC11446822; doi:10.1038/s41586-024-07953-5)
Supplement: Supplementary file 6 — CRF and ERF predictions for individual TmY4 and TmY9 cells. Analogous to Supplementary Data 3, but for TmY target types. Shown are the top four monosynaptic pathways, the strongest pathway passing through each of the top ten intermediary types (ranking from Extended Data Fig. 7), and the trisynaptic pathway Tm1–TmY–Dm3–TmY (see the section entitled Prediction of spatial normalization). [file 41586_2024_7953_MOESM6_ESM.zip › DataS4/TmY9qΓèÑ/720575940630884370.pdf]

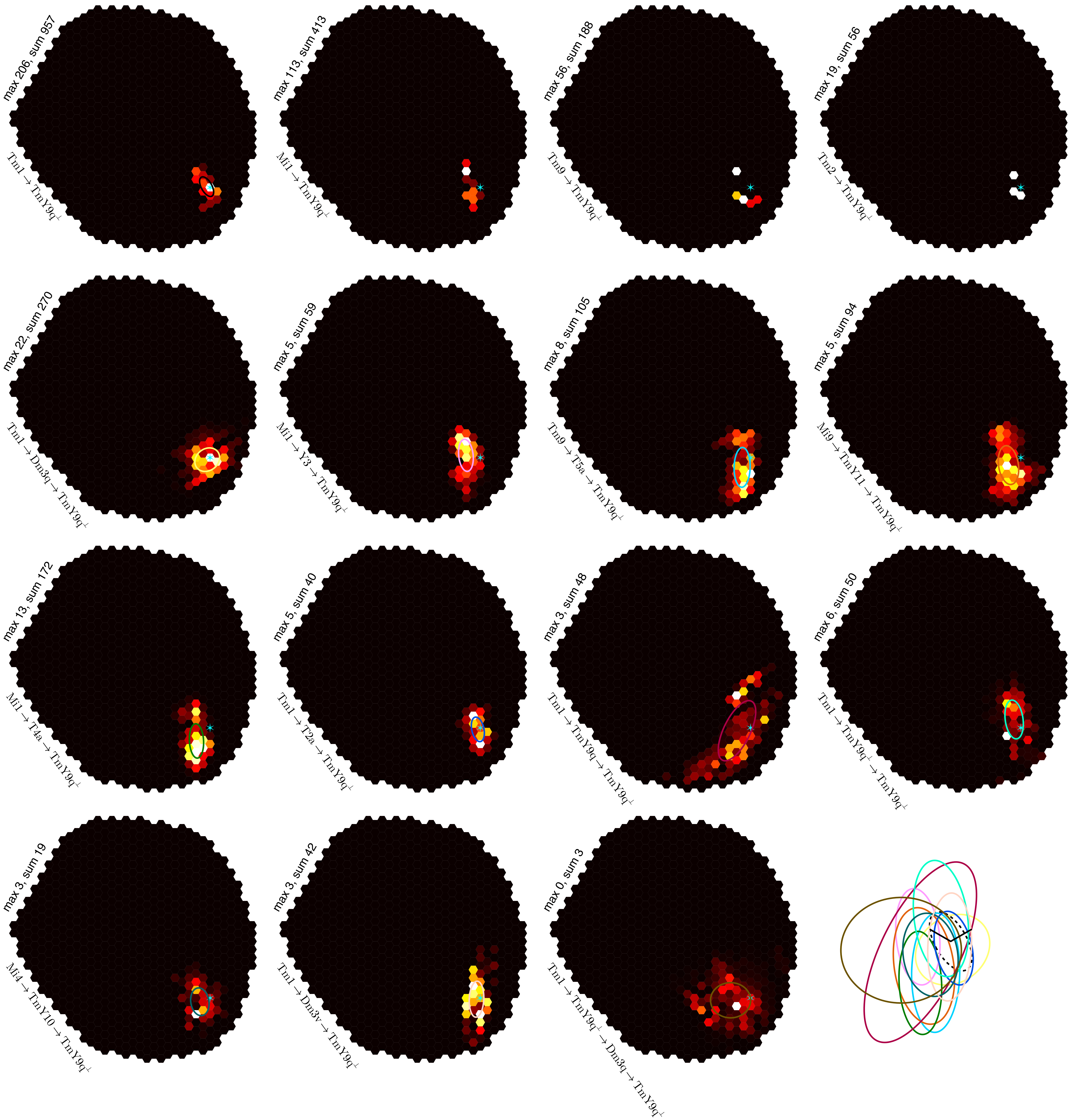

max 206, sum 957
Tm1 → TmY9q⊥
max 113, sum 413
Mi1 → TmY9q⊥
max 56, sum 188
Tm9 → TmY9q⊥
max 19, sum 56
Tm2 → TmY9q⊥
max 22, sum 270
Tm1 → Dm3q → TmY9q⊥
max 5, sum 59
Mi1 → Y3 → TmY9q⊥
max 8, sum 105
Tm9 → T5a → TmY9q⊥
max 5, sum 94
Mi9 → TmY11 → TmY9q⊥
max 13, sum 172
Mi1 → T4a → TmY9q⊥
max 5, sum 40
Tm1 → T2a → TmY9q⊥
max 3, sum 48
Tm1 → TmY9q → TmY9q⊥
max 6, sum 50
Tm1 → TmY9q⊥ → TmY9q⊥
max 3, sum 19
Mi4 → TmY10 → TmY9q⊥
max 3, sum 42
Tm1 → Dm3v → TmY9q⊥
max 0, sum 3
Tm1 → TmY9q⊥ → Dm3q → TmY9q⊥
